# Recombination Drives Evolution of the *Clostridium difficile* 16S-23S rRNA Intergenic Spacer Region

**DOI:** 10.1371/journal.pone.0106545

**Published:** 2014-09-15

**Authors:** Sandra Janezic, Alexander Indra, Thomas Rattei, Thomas Weinmaier, Maja Rupnik

**Affiliations:** 1 National Laboratory for Health, Environment and Food, Maribor, Slovenia; 2 Austrian Agency for Health and Food Safety (AGES), Vienna, Austria; 3 Faculty of Life Sciences, University of Vienna, Vienna, Austria; 4 Faculty of Medicine, University of Maribor, Maribor, Slovenia; 5 Centre of Excellence for Integrated Approaches in Chemistry and Biology of Proteins, Ljubljana, Slovenia; Institute Pasteur, France

## Abstract

PCR-ribotyping, a typing method based on size variation in 16S-23S rRNA intergenic spacer region (ISR), has been used widely for molecular epidemiological investigations of *C. difficile* infections. In the present study, we describe the sequence diversity of ISRs from 43 *C. difficile* strains, representing different PCR-ribotypes and suggest homologous recombination as a possible mechanism driving the evolution of 16S-23S rRNA ISRs. ISRs of 45 different lengths (ranging from 185 bp to 564 bp) were found among 458 ISRs. All ISRs could be described with one of the 22 different structural groups defined by the presence or absence of different sequence modules; tRNA^Ala^ genes and different combinations of spacers of different lengths (33 bp, 53 bp or 20 bp) and 9 bp direct repeats separating the spacers. The ISR structural group, in most cases, coincided with the sequence length. ISRs that were of the same lengths had also very similar nucleotide sequence, suggesting that ISRs were not suitable for discriminating between different strains based only on the ISR sequence. Despite large variations in the length, the alignment of ISR sequences, based on the primary sequence and secondary structure information, revealed many conserved regions which were mainly involved in maturation of pre-rRNA. Phylogenetic analysis of the ISR alignment yielded strong evidence for intra- and inter-homologous recombination which could be one of the mechanisms driving the evolution of *C. difficile* 16S-23S ISRs. The modular structure of the ISR, the high sequence similarities of ISRs of the same sizes and the presence of homologous recombination also suggest that different copies of *C. difficile* 16S-23S rRNA ISR are evolving in concert.

## Introduction

*Clostridium difficile*, an anaerobic, sporogenic bacterium, is one of the most important pathogens causing health care-associated infections. The spectrum of disease ranges from mild diarrhea to colitis and to sometimes fatal pseudomembranous colitis. Although, hospitalization and antibiotic usage are still considered as the main risk factors for *C. difficile* infection (CDI), affecting mainly elderly patient (>65 years), community-acquired infection is being increasingly reported [1]–[3]. The incidence and severity of CDI has increased dramatically since 2004, partially due to the emergence of more virulent strains (i.e. PCR-ribotypes 027, 078, 017, 053) [4]. Since then, small and large outbreaks are constantly being present in hospital environment [5]–[9].

A diverse set of molecular typing techniques has been used for molecular epidemiological studies of CDI, with PCR-ribotyping being the most popular method. PCR-ribotyping targets the intergenic spacer region (ISR) between the 16S and 23S rRNA genes [10]. Like in many other bacteria, several copies of the rRNA operon are present in *C. difficile* genome [11]. The 16S-23S rRNA ISRs of *C. difficile* differ in length and PCR amplification of ISRs with only a single primer pair results in a pattern of bands (ranging from ≈ 200 – 700 bp) which is unique for a specific PCR-ribotype. In *C. difficile*, the size variability of ISRs seem to be greater than in other bacteria which is reflected in the good discriminatory power of PCR-ribotyping [10], [12].

The rRNA genes in bacterial ribosomal operons are generally organized in the order of 16S-23S-5S rRNA and the individual genes are separated with the intergenic spacer regions (ISR) also called internal transcribed spacers (ITS). The spacer between the 16S and 23S rRNA has been studied the most and, due to various degrees of variability in different species, can be used either for typing, identification or phylogeny studies [13]–[19].

To date, only two groups have studied the variability of 16S-23S rRNA ISR in *C. difficile*. Sadeghifard *et al.* showed that ISRs have a mosaic structure and can differ in length as well as sequence [20]. On the other hand, Indra *et al*. showed that ISRs have a very uniform structure composed of tRNA gene (which is present or not) and different combinations of spacers of variable lengths separated by 9 bp direct repeat. They proposed two mechanisms that could be responsible for the ISR length variations; slipped-strand mispairing and/or homologous recombination [21].

In the present study, we describe the ISR sequence diversity and explore homologous recombination as a possible mechanism driving the evolution of *C. difficile* 16S-23S rRNA ISRs. Furthermore, we used the ISR sequence information to look for type or lineage specific markers within the ISRs which would be appropriate and have enough discrimination for sequence based typing.

## Results and Discussion

**Figure 1:**
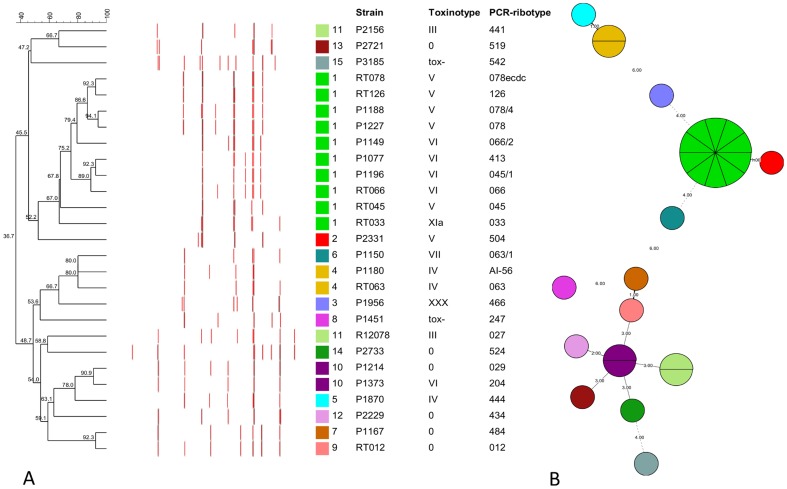
Clustering of *C. difficile* PCR-ribotypes. (A) Clustering of PRC-ribotypes based on fingerprinting profiles generated by capillary gel electrophoresis-based PCR-ribotyping. Dendrogram is color coded according to MLST type. The exact lengths of the bands, representing the 16S-23S rRNA intergenic spacer regions are given in Table S1. (B) Minimum spanning tree of MLST results showing relatedness of PCR-ribotypes. Each circle represents one sequence type (ST) and is subdivided into sectors corresponding to the number of PCR-ribotypes represented with this ST. The numbers between circles represent number of differing loci between the STs.

We analyzed 16S-23S rRNA ISR sequences of 43 *C. difficile* strains (Table 1). Twelve strains were either studied previously or had complete genome available [11], [20]–[23]. Additional thirty-one strains representing 27 different PCR-ribotypes were selected based on the similarity of banding patterns generated by capillary gel electrophoresis-based PCR-ribotyping (Figure 1a, Table S1). After screening the profiles of 136 PCR-ribotypes that were available at the time, we selected a subset of PCR-ribotypes that had similar banding patterns, clustering with PCR-ribotype 078 (these were considered to be more genetically related), and a subset of PCR-ribotypes that had very different banding patterns (considered to be genetically unrelated) (Figure 1a). Genetic relatedness of strains with a similar PCR-ribotyping profile has been suggested previously by Kurka *et al*. [24]. They compared 14 standard marker genes from 21 different PCR-ribotypes and showed that certain strains (e.g. PCR-ribotypes 078, 126 and 033) always clustered together indicating relatedness. We confirmed genetic relatedness of selected strains with multilocus sequence typing (MLST) and strains that had similar banding patterns by PCR-ribotyping were more likely to also cluster together with MLST (Figure 1b).

**Table 1:** Overview of *C. difficile* strains/genomes included in the analysis.

Strain	PCR-ribotype	Toxinotype	Host	Remarks
P2156	441	III	Human	
RT063	063	IV	Human	Reference RT strain^1^
RT066	066	VI	Human	Reference RT strain^1^
P2331	504	V	Human	
P1956	466	XXX	Human	
P1870	444	IV	Human	
P1451	247	TOX-	Human	
P2229	434	0	Human	
RT012	012	0	Human	Reference RT strain^1^
RT078	078ecdc	V	Human	Reference RT strain^1^
P2733	524	0	Human	
P3185	542	TOX-	Human	
RT033	033	XIa	Human	Reference RT strain^1^
RT045	045	V	Human	Reference RT strain^1^
P1373	204	VI	Human	
P1227	078	V	Calf	
P1214	029	0	Human	
RT126	126	V	Human	Reference RT strain^1^
ZZV08-1263	126	V	Human	
P1077	413	VI	Human	
P1180	AI-56	IV	Human	
P2721	519	0	Human	
P1167	484	0	Human	
ZZV08-961	045	V	Pig	
ZZV08-958	045	V	Pig	
SE866	045	V	Human	
P1150	063/1	VII	Human	
P1188	078/4	V	Human	
P1196	045/1	VI	Horse	
P1149	066/2	VI	Human	
R12078	027	III	Human	
CD196	027	III	Human	
R20291	027	III	Human	
630	012	0	Human	

The 16S -23S rRNA intergenic spacer regions (ISRs) from strains CD196 (NC_013315.1), R20291 (NC_013316.1) and 630 (NC_009089.1) were obtained from published *C. difficile* genomes. PCR-ribotypes were identified with WEBRIBO (http://webribo.ages.at/). We have included also 78 ISR sequences from 9 different strains (A, B, ATCC43593, 001, 027, AI5, 053, 078 and 176) analyzed in two previous publications [20], [21].

1 Leiden/Leeds reference PCR-ribotyping strain

### Length polymorphism of 16S-23S rRNA ISRs

With cloning and sequencing of amplified ISRs from 31 strains, we obtained 350 sequences. For each fragment (amplified ISR from all 27 PCR-ribotypes) at least one ISR was sequenced (1 – 60 clones per fragment of particular size as seen on banding patterns produced by capillary gel electrophoresis). In addition, we included 30 ISR sequences obtained from published *C. difficile* genomes of strains CD196 (NC_013315.1), R20291 (NC_013316.1) and 630 (NC_009089.1) and 78 ISRs from 9 different strains from two previous publications [20], [21]. Forty-five different sizes were recognized among the 458 ISR sequences, ranging from 185 to 564 bp.

### ISR structure - a new spacer

**Figure 2:**
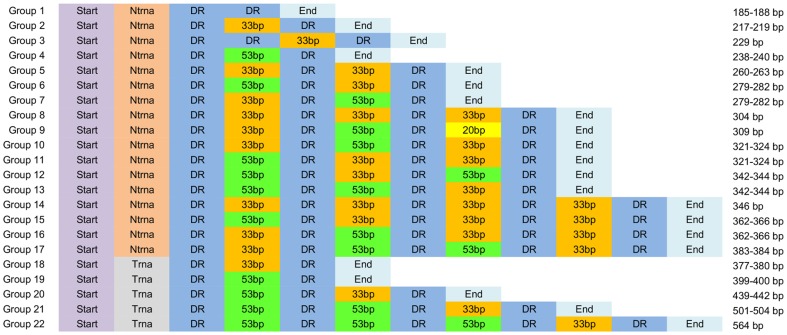
Schematic representation of the modular structure of *C. difficile* 16S-23S rRNA intergenic spacer region. Start (29 bp) – 5' end of the ISR sequence; Ntrna (26 bp) – part of the ISR without a gene for tRNA^Ala^; Trna (186 bp) – part of the ISR with a gene for tRNA^Ala^; DR – 9 bp long direct repeat; 53 bp, 33 bp, 20 bp – spacers of 53 bp, 33 bp and 20 bp, respectively and End (103–114 bp) is the 3' end of the ISR. The size of each structural group is given on the right. The inverse spacer arrangements can be seen in groups 6 and 7, 10 and 11, 12 and 13 and in groups 15 and 16.

Based on visualization of previously described modules within *C. difficile* ISRs [21], all ISR sequences were manually grouped into 22 different structural groups (Figure 2), 14 of which have been described previously [21]. In general, the variations in ISRs were due to the different number and organization of building blocks. All ISRs had the same basic structure beginning with a start sequence of 29 bp, followed by either 26 bp (in ISRs without tRNA^Ala^) or 186 bp (in ISRs with tRNA^Ala^). Next was a 9 bp direct repeat followed by different combinations of spacers of three different lengths (33, 53 and 20 bp) where spacers were separated by a 9 bp direct repeat. At the end of the ISR sequence was the last direct repeat followed by a 103–114 bp end sequence (Figure 2). Two of the spacers (33 and 53 bp) have been described already [21], while the third one (20 bp) was found in an ISR of 309 bp in PCR-ribotype 012 (two different isolates, RT012 and CD630). The shortest sequence (185 bp) was composed only of the start sequence (without tRNA gene) followed by two direct repeats and the end sequence (group 1, Figure 2). Only one ISR (229 bp, group 3; found in PCR-ribotype 542) did not follow the general structure and had two direct repeats after the start sequence followed by a spacer of 33 bp and direct repeat before the end sequence.

Table 2 lists the number of different sequence variants and consensus sequence for each building block. When looking for sequence variations only the representatives of non-redundant sets (n = 95) were taken into account. All sequence variants of ISR building blocks and sequence logos showing conserved and variable sites are listed in Table S2 and Figure S1.

**Table 2:** Consensus sequence and number of sequence variants found in ISR building blocks.

Building block	Sequence length	Nr. of sequences	Nr. of variants	Consensus sequence
**Start**	29 bp	95	4	AAGGAGAATTACCTACTGTTTAATTTTGA
**Ntrna**	26 bp	72	5	GGGTTCGTTTTTACGAATACTCAAAA
**Trna**	186 bp	23	11	AAGTTCTTTACGAACTTTATATATGGGGGTGTAGCTCAGTTGGGA GAGCACTTGCCTTGCAAGCAAGGGGTCAGGAGTTCGACTCTCCTC ATCTCCACCATTTAAGAGTATATTACTTAAATCTTTGATTTACTTAG TAGCCTCTTACAATGCACTTATAGCTTAAATTTATACAAGCTTTGTGCG
**DR**	9 bp	335	8	TTAGCACTT
**20 bp**	20 bp	3	1	TAAGCAACGGAATTTATTCG
**33 bp**	33 bp	132	42	TAAGCAACGGAATTTATTCGTTGGCGCTGTGCT
**53 bp**	53 bp	98	35	TAAGCAACAGAATAAACTGAACGCATGTGAAGTTTGTTTGTTG GCGCTGTGCG
**End**	114 bp	95	33	TGAAAACTGCATATATATATTTAGTGATATGACATCTAATTTGTA ATATATAAAGCTGATAACTTTTTAAAATTATCGAAGTTGATAGCT TCTAATCTATCAAACCTTTTTAAC

Only the representatives (n = 95) of non-redundant sets were used to calculate the consensus sequence and to determine the number of sequence variants.

### ISR structural group coincide with the sequence length

In the majority of cases, the ISRs of the same length were represented with only one structural group. Four exceptions were found where ISR sequences of the same or similar lengths (±3 bp) were represented by two different structural groups; these included ISRs of 279–282 bp represented by structural groups 6 and 7, ISRs of sizes 321–324 bp represented by structural groups 10 and 11, ISRs of sizes 342 bp and 344 bp represented by groups 12 and 13, and ISRs of 362–366 bp represented by structural groups 15 and 16. The only difference between the two groups with the ISRs of the same lengths was the inverse arrangements of spacers of 33 bp and 53 bp (Figure 2). The inverse arrangement of spacers and their similar sequence can result in incorrect hybridization of different ISRs influencing the correct PCR-ribotype designation when using electrophoresis in non-denaturing conditions for separation of amplified ISRs. This was observed previously for PCR-ribotype AI-5 that was differentiated from PCR-ribotype 001 with classical agarose gel electrophoresis-based PCR-ribotyping by a single band, and had an identical banding pattern with capillary gel electrophoresis-based PCR-ribotyping [21]. Heteroduplex formation in 16S-23S rRNA ISR has also been described for cyanobacteria [25].

### Presence of tRNA genes in the ISRs

16S-23S rRNA ISRs can have different tRNA genes. In the majority of bacteria only one or two tRNA genes are present in the ISR [26], but some bacteria can have 3 or even 4 different tRNA genes in a single ISR [27]. In *C. difficile* only one tRNA gene was present in ISRs and it was always tRNA^Ala^. The occurrence of tRNA^Ala^ in *C. difficile* ISRs has been described by others [20], [21], [26], [27]. The tRNA^Ala^ gene was always located at the same site, as a part of the 186 bp sequence located between the start sequence and first direct repeat (Figure 2). When we searched for tRNA genes in published *C. difficile* genomes, the tRNA^Ala^ gene was present only in the ISR and not elsewhere in the genome. This explains why each strain had at least one ISR that contained a tRNA^Ala^ gene.

### ISR sequence similarities

Single PCR-ribotype yielded from 4 to 11 fragments ranging in size from 234 to 609 bp after separation of the amplified ISRs with capillary gel electrophoresis (Figure 1a, Table S1). Some of the fragments were present only in a single strain (e.g. 609 bp in PCR-ribotype 524 and 279 bp in PCR-ribotype 542) and others in several strains. One fragment of approximately 329 bp (±2 bp) was present in all but one strain (PCR-ribotype 504) included in the analysis (Figure 1a, Table S1).

Sadeghifard *et al*. concluded that ISRs of similar lengths could have noticeably different sequence, within and between different strains [20]. Furthermore, they suggested that this high degree of diversity is not apparent when just the length polymorphisms are considered. Our data contrast with those results and show that nucleotide sequences of ISRs with the same or similar length (± 3 bp) are very similar (sequence identity > 92 to 100%; only the 95 representatives of non-redundant sets were taken into account). The only exceptions were found in ISRs of 279–282 bp, 321–324 bp, 342–344 bp and 362–366 bp where the similarities were lower than expected; 80,2%, 82,4%, 83,0% and 83,6%, respectively (Supporting information S1). The explanation for this finding is related to the inverse spacer arrangements (Figure 2). ISRs with only one or both variants (inverse arrangement of spacers) were found within a single strain. That the ISR sequences are really very similar is demonstrated also by the unsupervised clustering. All 458 ISRs could be grouped into only 95 non-redundant sets (with 99% sequence identity and 99% alignment coverage).

Some of the ISRs with identical nucleotide sequences differed in length by only 2 bp (e.g. 502 and 504, 280 and 282, 264 and 266…). The length difference in these sequences was always additional or missing di-nucleotide (AT or TA) within the stretch of AT/TA repeats (2–4) at the 3' end of the ISR (Figure S2). Clarke *et al*. showed that a high error rate at repeat sequence motif could be introduced by PCR when amplifying mono- and di-nucleotide sequence motifs [28]. Whether this 2 bp difference is real and there are two different alleles present in the genome or it is just a PCR artefact needs to be clarified.

### Absence of PCR-ribotype specific sequences for possible sequence based typing

The length polymorphism in the *C. difficile* 16S-23S rRNA ISR has been successfully used for fingerprint based PCR-ribotyping, which has become the method of choice for typing of *C. difficile* worldwide. However, as with other non-sequence based methods, the disadvantage of the method is still poor inter-laboratory comparability and exchangeability of results. One of the goals of the present work was to develop a sequence based method, utilizing the ISR or parts of the ISR that would enable us to discriminate between strains at least on the level of PCR-ribotyping. But due to the highly uniform ISR sequence structure there were no regions appropriate to position primers which would enable us to specifically amplify a single ISR. Our data also indicate that the nucleotide sequences of ISRs are not variable enough to distinguish between strains of different PCR-ribotypes. ISRs with identical nucleotide sequence were found in related and unrelated PCR-ribotypes. Only a few ISRs were strain specific (in PCR-ribotypes 066/2, 524 and 542) however, it is very likely that these three ISRs are present in some other PCR-ribotypes that were not included in this study.

Despite the lack of PCR-ribotype specific markers, we were able to use the information from the *C. difficile* ISR sequence to modify the primers currently used for PCR-ribotyping and to describe an approach that enables direct, culture independent PCR-ribotyping [29].

### Secondary structure based alignment of ISR

The modular structure and extensive variation in sequence length make the alignment of ISR sequences unreliable. Therefore, we used the primary and secondary structure information of ISRs to align 95 representatives of non-redundant sets (Figure S2). The automatic alignment was then checked manually to verify it and correct misaligned sequences. Secondary structure information can be used to improve the alignment and tree reconstruction of the ribosomal rRNA sequences [19], [30], [31].

Despite large variations in the length of ISRs, the aligned sequences showed parts of highly conserved regions (Figure S2), especially the 5′ end (first 29 bp) and 3′end (last 112 bp) of ISRs that showed very little sequence variation and were highly conserved between all strains. These regions are thought to be involved in maturation of pre-rRNA [26], [32]. To test this we built secondary structure models to illustrate the interactions between these regions. The genomic sequences used for the secondary structure calculations contained parts of approximately 100 bp of the region upstream of 16S rRNA, the 16S-23S ISR, approximately 50 bp at the 5′ end of 23S-5S ISR and tRNA^Ala^ when present. Genes coding for 16S and 23S rRNA were excluded from the calculations. Predicted secondary structures were determined for five rRNA operons (two without the tRNA^Ala^ gene within the ISR and three with the tRNA^Ala^ gene) of *C. difficile* CD630 strain [11]. In all five rRNA operons, the 5′ end of the ISR was base paired with the region upstream of the 16S, forming a stem that carried the 16S rRNA gene, and the 3′ end of the ISR was paired with the 23S-5S ISR to form a stem that carried the 23S rRNA gene (Figure S3). Direct repeats and spacers, which were identified in the primary structure, did not seem to be conserved in the secondary structure (Figures S2 and S3).

### Role of recombination in evolution of *C. difficile* 16S-23S rRNA ISR

**Figure 3:**
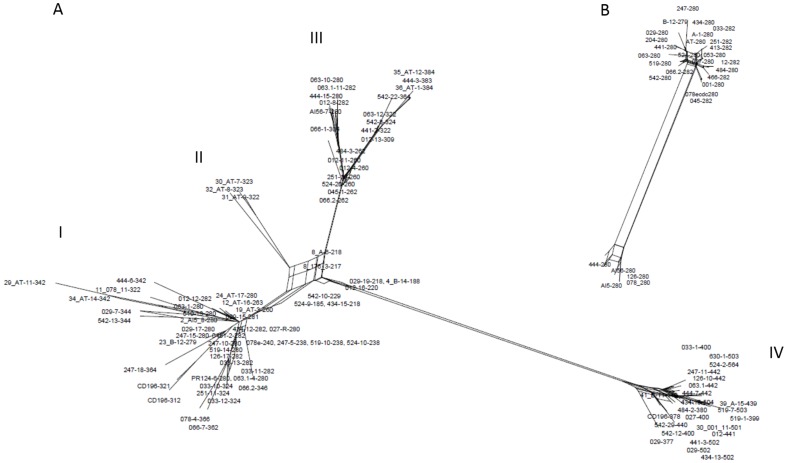
Indication of homologous recombination in *C. difficile* 16S-23S rRNA intergenic spacer region. (A) Phylogenetic network constructed for 95 representative ISR sequences from 43 different *C. difficile* strains. (B) Phylogenetic network of 29 ISR sequences (279–282 bp) from 29 different strains of *C. difficile*. Only one ISR per strain was included in the analysis. Box-like branches seen on both figures indicate relative support for alternative relationships among ISRs, probably resulting from homologous recombination that was subsequently confirmed by statistical analysis. In groups I, II and III the ISRs without a gene for tRNA^Ala^ are clustered and in group IV the ISRs with a tRNA gene.

As discussed above, a given ISR is a mosaic of variable blocks and this specific structure of the ISR could be the result of recombination between different rRNA operons. Homologous recombination has been described as one of the possible mechanisms responsible for variation within rRNA operons in other species, such as the exchange of ISRs between rRNA operons detected in *Vibrio cholerae* [33] and rearrangements of sequence blocks within the ISR as described for *Haemophilus parainfluenzae* [34]. Other features that suggest homologous recombination as a possible mechanism of variation in the *C. difficile* 16S-23S rRNA ISR are the absence of strain specific ISRs or sequence blocks, tRNA genes not being present in all ISRs, the inverse spacer arrangements and the observation of identical ISRs in unrelated strains. Two mechanisms that could be responsible for the length variations in *C. difficile* ISRs have been proposed; slipped-strand mispairing and/or homologous recombination [21]. To further explore the role of homologous recombination in *C. difficile* ISRs the aligned sequences were analyzed with SplitsTree software. A phylogenetic network was constructed for the 95 representative ISR sequences grouping the sequences into 4 groups (I – IV) (Figure 3A). The ISRs without a gene for tRNA^Ala^ clustered in groups I, II and III and the ISRs with a tRNA gene present were in group IV only. The box-like topology of the network (ISRs are connected by several branches), especially within the groups and between groups I, II and III, represents conflicting phylogenetic signals indicating that recombination events might have contributed to the evolution of ISRs (Figure 3A). As conflicting signals can be explained by recombination or homoplasies (parallel mutations) three statistical tests; PHI (pairwise homoplasy index), maximum chi-squared and NSS for detecting the presence of recombination were applied [35]–[37]. All three tests clearly supported recombination (p = 0.0). The phylogenetic network of 29 ISR sequences (279–282 bp) from 29 different strains of *C. difficile*, only one ISR per strain was included in the analysis (Figure 3B), also showed conflicting signals indicating the possibility of inter-chromosomal recombination (PHI test; p = 0.0013; 55 informative sites).

Since our results suggest that the evolution of *C. difficile* 16S-23S rRNA ISRs is mainly influenced by recombination, ISRs would not be considered as appropriate marker for study of phylogenetic relationships of *C. difficile* strains. However, comparison of similarities between PCR-ribotypes showed that PCR-ribotypes that have similar banding patterns are related, as shown by MLST analysis (Figure 1) and the whole genome comparisons shown by others [24], suggesting that despite recombination in ISR PCR-ribotyping is an appropriate method to group *C. difficile* strains. This could indicate that recombination within ISR regions occurs with slow rate and in parallel to other evolutionary changes within *C. difficile* species. Furthermore, Valiente *et al*. recently described 3 different *C. difficile* PCR-ribotypes that have probably evolved from the 027 lineage and which banding patterns differ from the 027 in just one or two bands (i.e. 176 and 198 differing from 027 by a single band and 244 differing by two bands, one absent and one additional band) [38]. It is therefore possible that gain or loss of ISRs of particular sizes is due to rearrangements of building blocks within the ISRs, or even entire ISRs or rRNA operons, via the process of homologous recombination or possibly also slipped-strand mispairing, another proposed mechanism responsible for size variations in 16S-23S rRNA ISR [21].

All these features described for the 16S-23S rRNA ISRs are consistent with the highly mobile and mosaic *C. difficile* genome with up to 11% of the genome comprising horizontally acquired genetic elements [11]. Moreover, studies based on whole genome comparisons have shown that beside mobile elements homologous recombination has also played an important role in the evolution of *C. difficile* [23]. This was recently demonstrated by Brouwer and colleagues [39] who showed that large sequence blocks (up to 270 kbp) can be transferred between *C. difficile* strains and subsequently being integrated into the recipient genome.

Our results indicate that the evolution of *C. difficile* 16S-23S rRNA intergenic spacer region (ISR) is mainly influenced by recombination (intra- and inter-genomic). The modular structure of the ISRs, the high sequence similarities of ISRs of the same sizes and evidence of homologous recombination also suggest concerted evolution (homogenization among different loci in multigene families) of 16S-23S rRNA ISRs. Despite the strong evidence of recombination in ISRs our data show that PCR-ribotyping is an appropriate method to group *C. difficil*e strains, as strains with similar banding patterns were related by MLST.

## Materials and Methods

### Isolates and genomes

Thirty one isolates of 27 different PCR-ribotypes were selected from two strain collections (Table 1). Ten strains were from the National Laboratory for Health, Environment and Food (NLZOH) strain collection and 21 strains were from the Austrian National reference Centre for *C. difficile*, Vienna, Austria. Three isolates originated from animals, two from piglets from two Slovenian farms and one from a calf from Canada. The remaining 28 isolates were from humans from different geographical locations. ISRs from 3 sequenced *C. difficile* genomes were also included in the analysis; CD196 (NC_013315.1), R20291 (NC_013316.1) and 630 (NC_009089.1) [11], [22], [23]. For one strain (PCR-ribotype 027) the ISRs were obtained from sequenced genome (CD196) and with cloning of amplified ISRs. We have included also 78 ISR sequences from 9 different strains; A, B, ATCC43593, 001, 027, AI5, 053, 078 and 176 analyzed in two previous publications [20], [21].

### Molecular characterization

DNA was extracted using a MagNA Pure Compact instrument (Roche Diagnostics) according to the manufacturer's recommendations.

All strains were characterized by toxinotyping involving amplification and enzymatic restriction of PCR fragment A3 of the *tcdA* gene and PCR fragment B1 of the *tcdB* gene as described previously [40]. Absence of the PaLoc was confirmed by PCR amplification of the 115 bp sequence integrated at this site [41].

PCR-ribotyping was performed as described before [42]. Amplified fragments were analyzed with a 310 Genetic Analyzer (Applied Biosystems) with a 41 cm capillary in a POP4 gel. A TAMRA ladder 50–625 bp (Chimerx) was used as a size marker. Injection of samples was done at 5 kV over 5 s, and the total running time was 28 min at 15 kV. The size of each peak was determined using Peakscanner software 1.0 (Applied Biosystems). Peaks were counted as bands when they showed at least 10% of the height of the highest peak of each run. PCR-ribotypes were identified with Webribo software (http://webribo.ages.at/). The peaks were also imported into BioNumerics software version 7.1 (Applied Maths) and the Dice coefficient and UPGMA clustering method were used to compare banding patterns. Optimization and position tolerance were set to 0 and 0.5, respectively.

For multilocus sequence typing (MLST) the scheme described by Lemee *et al*. [43], using seven housekeeping genes (*aroE*, *dutA*, *gmk*, *groEL*, *recA*, *sodA* and *tpi*), was used. After PCR amplification, products were purified with Exonuclease I and Shrimp alkaline phosphatase (Fermentas), following manufacturer's recommendations. Sequencing reactions were set up using BigDye Terminator v1.1 kit (Applied Biosystems) and PCR products were purified with Centri-Sep columns (Princeton Separations) before sequencing on an ABI 3130 (Applied Biosystems). Sequences with forward and reverse primers for all seven loci were imported into BioNumerics software version 7.1 (Applied Maths) and analyzed with the MLST plugin. Minimum spanning tree (MST) was used to present MLST data.

### Amplification of the 16S-23S rRNA intergenic spacer region (ISR)

The 16S-23S rRNA ISRs were amplified with the same primers used for PCR ribotyping; 16S (5′-GTGCGGCTGGATCACCTCCT) and 23S (5′-CCCTGCACCCTTAATAACTTGACC) [10]. PCR was carried out in a final volume of 50 µl containing 25 µl HotStar Taq Master Mix (Qiagen), 50 pmol of each primer and 1.5 µl of DNA. Amplifications conditions were as follows: an initial denaturation step of 15 min at 95°C, followed by 35 cycles of 1 min at 94°C for denaturation, 1 min at 57°C for annealing and 1 min at 72°C for elongation, plus 7 min at 72°C for a final elongation. The resulting amplification products were first checked by electrophoresis in 1.5% agarose gels and then purified with a MinElute PCR purification kit (Qiagen), following manufacturer instructions.

### Cloning and sequencing of 16S-23S rRNA ISR

The purified amplification products were cloned into the pDrive vector (PCR Cloning Plus kit; Qiagen) according to the manufactureŕs instructions. Plasmids were isolated from transformed overnight colonies using QIAprep Spin Miniprep kit (Qiagen).

Plasmids (1 µl) were checked for inserts by re-amplification using primers M13r and M13f. Sequencing of the clones with an insert was carried out using Genetic analyser ABI 3130 (Applied Bosystems) and BigDye Terminator v1.1 Cycle Sequencing Kit (Applied Biosystems). Sequencing reactions contained 2 µl of template DNA, 2 µl of BigDye Terminator v1.1, 1 µl of sequencing buffer and 1 µl of either primer M13f or M13r in a total volume of 10 µl. After the sequencing PCR, products were purified using Centri-Sep columns (Princeton Separations), according to the manufacturer's instructions.

The lengths of the individual ISRs obtained from direct cloning were compared to the PCR-ribotyping profile (all PCR amplified ISRs from a given genome). If not all ISRs were obtained with direct cloning of PCR products the remaining fragments were excised and purified from the agarose gel using a QIAquick Gel Extraction Kit (Qiagen). The purified products were then cloned and sequenced as described above.

### ISR sequence analysis

The sequence of the vector was trimmed from all sequences using the program CLC DNA workbench (CLC bio, Denmark) and BioEdit Sequence Alignment Editor [44]. The same two programs were also used for assembly of the sequences generated with forward and reverse primers. Consensus sequence and sequence logos were generated with Weblogo3 [45]. tRNAscan-SE was used to scan the genomes for tRNA genes [46].

To facilitate computational analysis, sequence clustering using CD-HIT was used to make non-redundant sequence sets with 99% sequence identity and 99% coverage [47]. Sequences of representatives of non-redundant sets are provided in FASTA format (Dataset S1). All representatives in the non-redundant datasets were aligned using LocARNA software using default parameters [48]. LocARNA first calculates the secondary structure for all ISR using RNAfold and then generates a multiple alignment that conserves as many structural features as possible.

The evolutionary relationships among the ISRs were constructed using the Neighbor-Net (part of the SplitsTree4 software) which illustrates the relationships between the ISR by constructing networks, instead of bifurcating phylogenetic trees, taking into account also recombination events that might have occurred during evolution. Three different statistics were used to test for recombination; PHI test (a pairwise homoplasy index) [36], [49], maximum chi-squared [37] and NSS [35], using the PhiPack software (http://www.maths.otago.ac.nz/∼dbryant/).

A secondary structure prediction of the primary rRNA transcript was constructed using the RNAfold web service [50]. To simplify the secondary structure model, the 16S, 23S and 5S rRNAs were deleted from the sequence.

## Supporting Information

Figure S1 **Sequence logos showing conserved and variable nucleotide sites in the ISR building blocks.**
(PDF)

Figure S2 **LocARNA alignment of ISRs with consensus sequence and conservation of the sequences.** All 95 representatives of non-redundant data set were included in the alignment.
(TIFF)

Figure S3 **Proposed secondary structure model of the RNA transcript of** ***C. difficile*** **rRNA operon.** The secondary structure shown in (A) represent the rRNA operon with the ISR without the tRNA^Ala^ gene and (B) with tRNA^Ala^ gene present. The 16S and 23S are represented by triangles. Direct repeats are marked with boxes.
(PDF)

Table S1 **Banding patterns of PCR-ribotypes included in the analysis.** Number and sizes of amplified ISRs (including primers) obtained for each PCR-ribotype using the sequencer based PCR-ribotyping.
(PDF)

Table S2 **List of sequence variants of ISR building blocks.** Only the 95 representatives of nun-redundant datasets were taken into account when looking at the variations in ISR sequence building blocks.
(PDF)

Dataset S1 **Representative 16S-23S rRNA ISR sequences of non-redundant sets (99% sequence identity and 99% sequence coverage).** Sequences are in Fasta format.
(TXT)

Supporting Information S1 **Sequence identity matrices of 95 representatives of non-redundant sets.**
(PDF)
