# Supplementary material for: Recombination Drives Evolution of the Clostridium difficile 16S-23S rRNA Intergenic Spacer Region
Source: PLoS One. 2014 Sep 15;9(9):e106545. doi: 10.1371/journal.pone.0106545 (PMC4164361; doi:10.1371/journal.pone.0106545)

Figure S1. Sequence logos showing conserved and variable nucleotide sites in the ISR building blocks.

Start

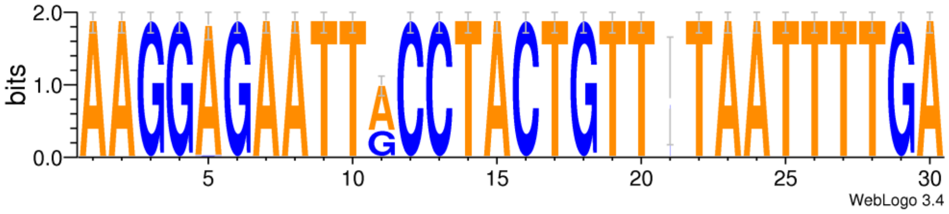

Ntrna

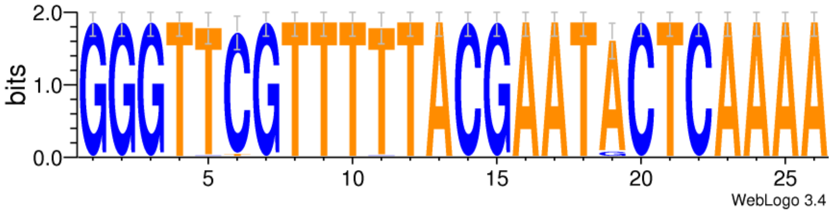

Trna

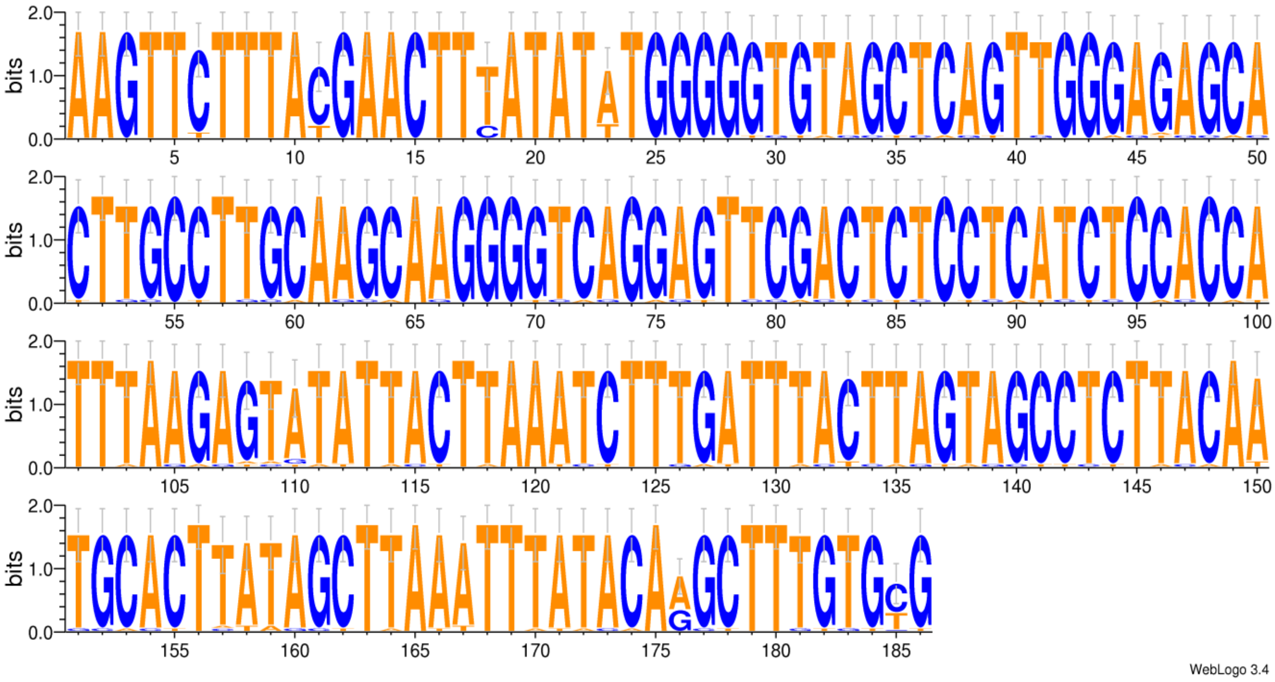

9 bp direct repeat (DR)

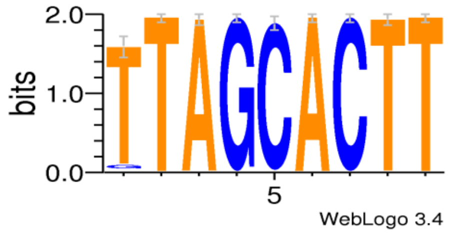

33 bp spacer

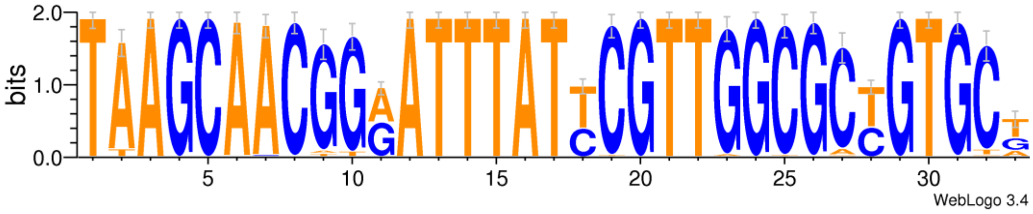

53 bp spacer

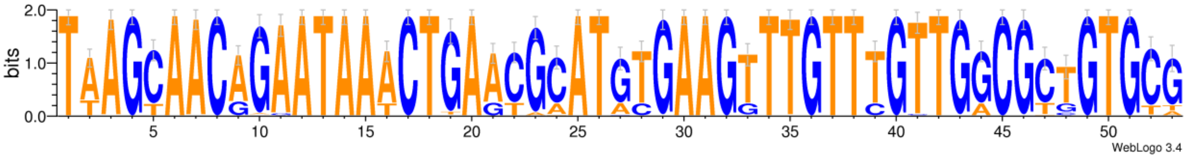

End

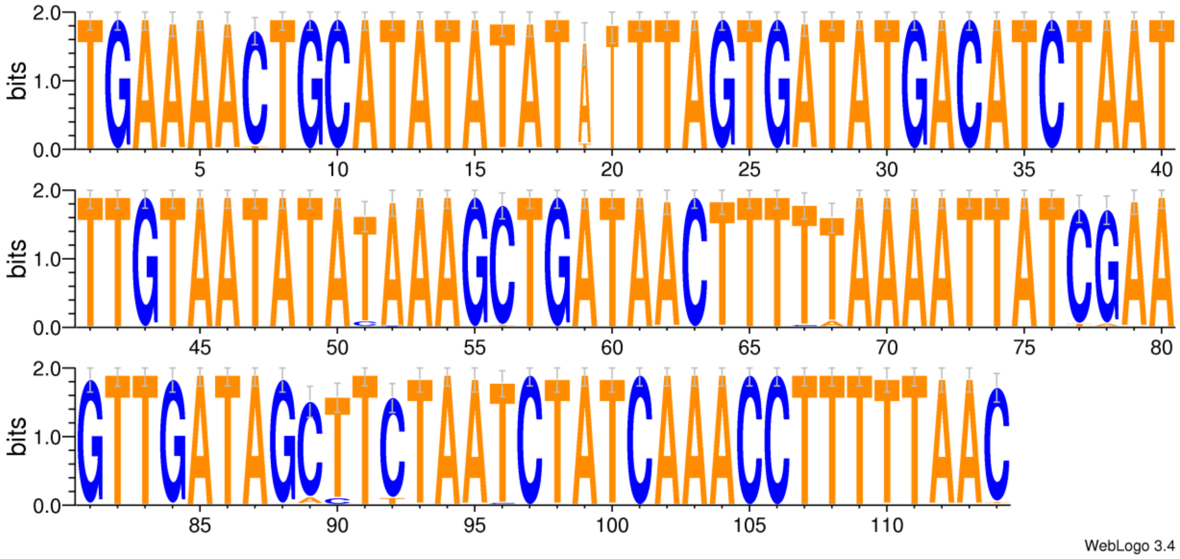

Supplement: Figure S1 — Sequence logos showing conserved and variable nucleotide sites in the ISR building blocks. (PDF) [file pone.0106545.s001.pdf]
