# Supplementary figures and images for: Recombination Drives Evolution of the Clostridium difficile 16S-23S rRNA Intergenic Spacer Region
Source: PLoS One. 2014 Sep 15;9(9):e106545. doi: 10.1371/journal.pone.0106545 (PMC4164361; doi:10.1371/journal.pone.0106545)

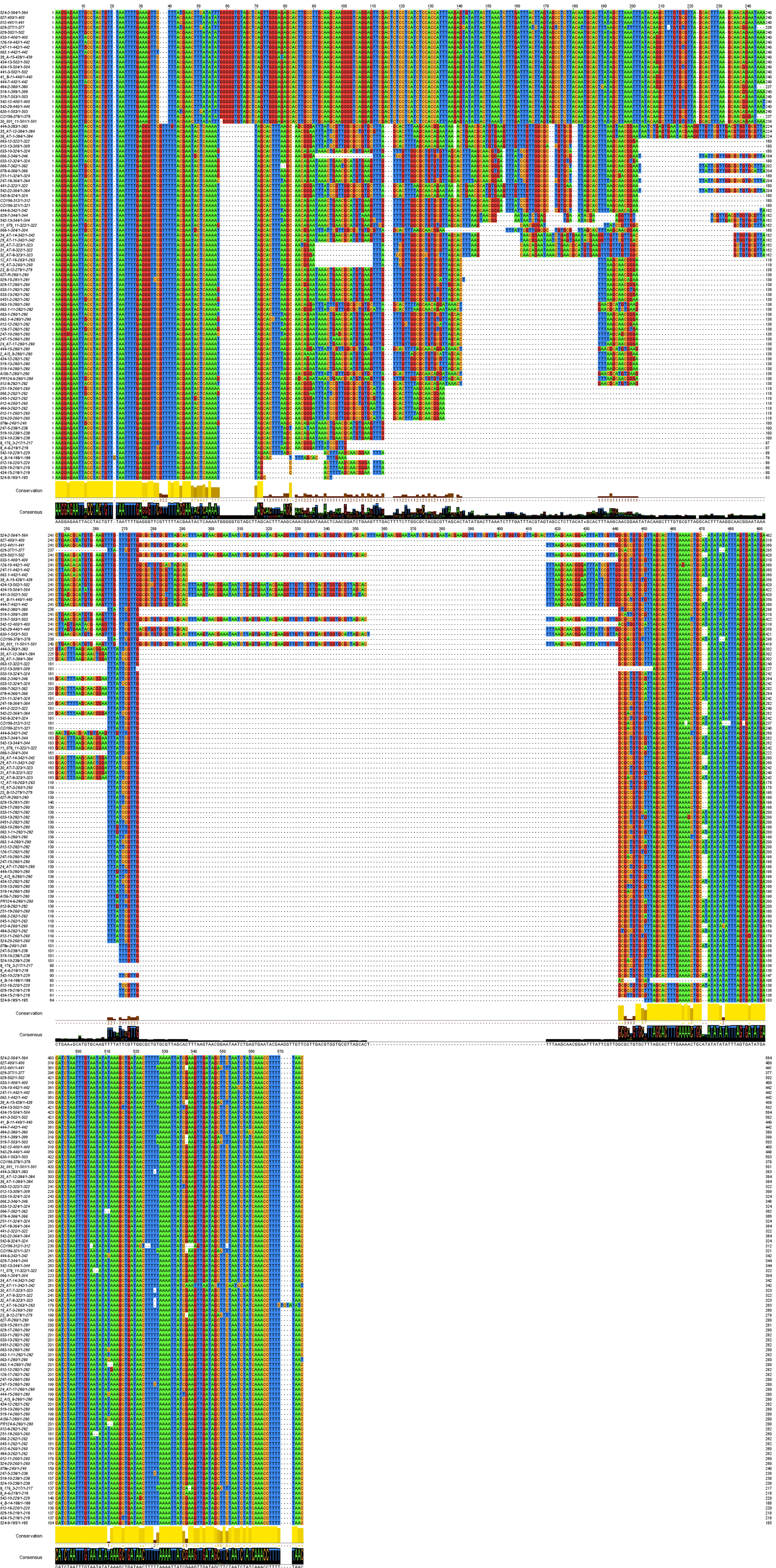

Supplement: Figure S2 — LocARNA alignment of ISRs with consensus sequence and conservation of the sequences. All 95 representatives of non-redundant data set were included in the alignment. (TIFF) [file pone.0106545.s002.tiff]
