# Supplementary material for: Recombination Drives Evolution of the Clostridium difficile 16S-23S rRNA Intergenic Spacer Region
Source: PLoS One. 2014 Sep 15;9(9):e106545. doi: 10.1371/journal.pone.0106545 (PMC4164361; doi:10.1371/journal.pone.0106545)

Figure S3. Proposed secondary structure model of the RNA transcript of *C. difficile* rRNA operon.

(A) ISR without the $tRNA^{Ala}$ gene and (B) ISR with $tRNA^{Ala}$ gene. The 16S and 23S rRNA are represented by triangles. Direct repeats are marked with boxes.

A

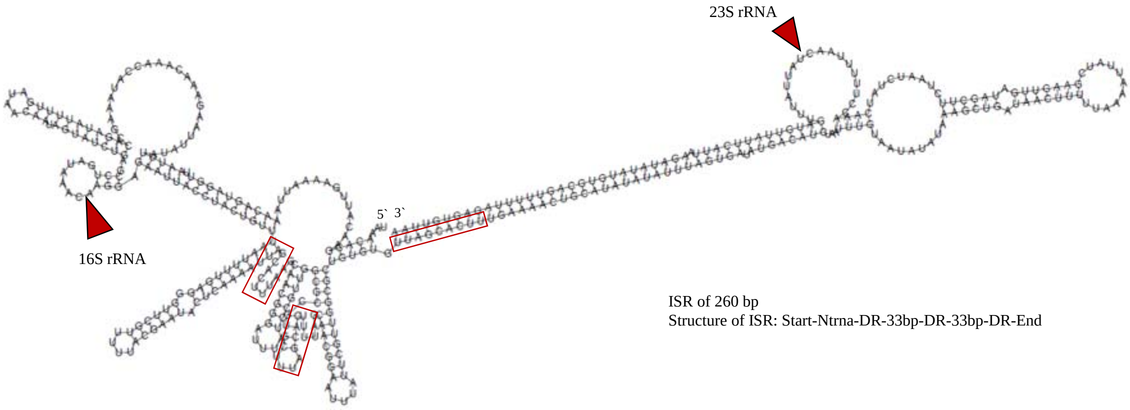

B

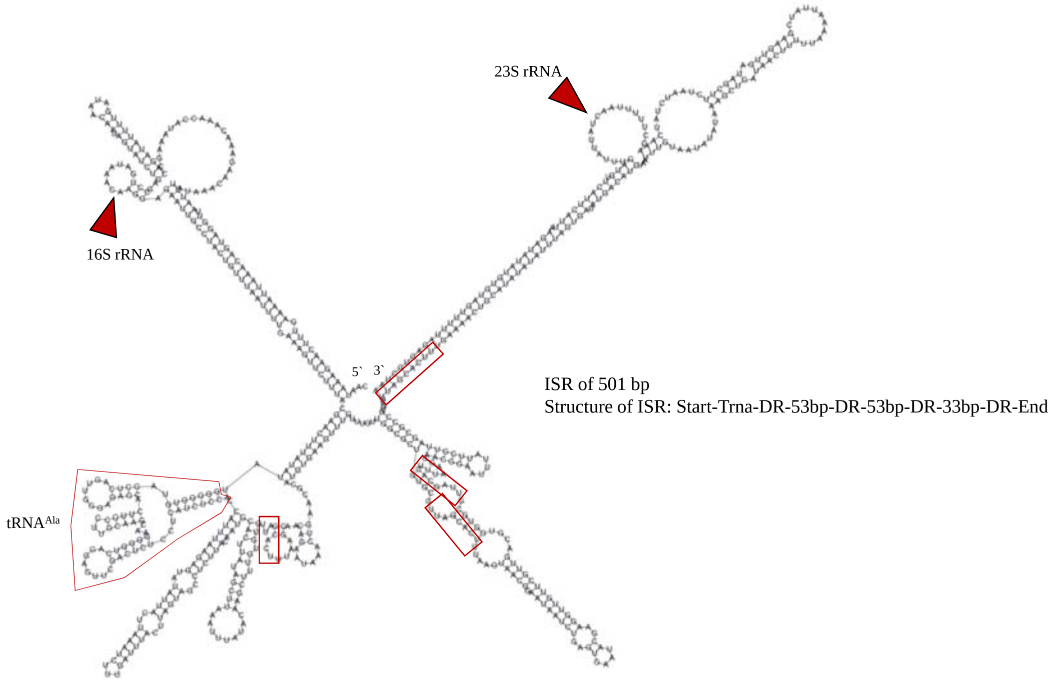

Supplement: Figure S3 — Proposed secondary structure model of the RNA transcript of C. difficile rRNA operon. The secondary structure shown in (A) represent the rRNA operon with the ISR without the tRNAAla gene and (B) with tRNAAla gene present. The 16S and 23S are represented by triangles. Direct repeats are marked with boxes. (PDF) [file pone.0106545.s003.pdf]
