# Supplementary material for: Recombination Drives Evolution of the Clostridium difficile 16S-23S rRNA Intergenic Spacer Region
Source: PLoS One. 2014 Sep 15;9(9):e106545. doi: 10.1371/journal.pone.0106545 (PMC4164361; doi:10.1371/journal.pone.0106545)
Supplement: Table S1 — Banding patterns of PCR-ribotypes included in the analysis. Number and sizes of amplified ISRs (including primers) obtained for each PCR-ribotype using the sequencer based PCR-ribotyping. (PDF) [file pone.0106545.s004.pdf]

Table S1. Banding patterns of PCR-ribotypes included in the analysis obtained by sequencer based PCR-ribotyping. Fragment lengths are in base pairs.

Strain<br>PCR-ribotype	P1227<br>078	P1373<br>204	P1451<br>247	P1870<br>444	P2156<br>441	P2229<br>434	P2733<br>524	P1214<br>029	P1149<br>066/2	RT078<br>078ecdc	RT126<br>126	RT012<br>012	R12078<br>027
Fragment 1	308,5	268,7	267,4	268,8	328,6	267,1	235,0	268,5	313,0	308,5	308,5	268,8	234,7
Fragment 2	329,0	328,5	286,9	328,8	372,7	268,8	268,5	328,4	329,0	329,1	329,1	310,9	268,4
Fragment 3	331,5	388,3	328,5	330,4	447,0	328,5	288,6	388,3	331,5	331,6	331,5	330,9	328,7
Fragment 4	373,7	488,2	411,5	389,7	551,5	388,2	308,5	428,1	373,0	375,1	372,9	359,1	330,7
Fragment 5	375,1	549,5	489,4	431,2		449,3	327,2	488,0	375,2	447,4	375,1	428,5	366,8
Fragment 6	417,0			489,1		549,5	329,1	549,5	387,3	491,4	447,4	488,1	427,0
Fragment 7	447,5						489,4		447,4		491,4	549,8	449,7
Fragment 8	491,5						550,5						549,5
Fragment 9							609,1						
Fragment 10													
Fragment 11													

Strain<br>PCR-ribotype	P1150<br>063/1	P1167<br>484	P1180<br>AI-56	P1188<br>078/4	RT033<br>033	RT045<br>045	RT063<br>063	RT066<br>066	P1077<br>413	P1196<br>045/1	P2331<br>504	P2721<br>519	P1956<br>446	P3185<br>542
Fragment 1	268,8	268,8	328,7	308,3	268,8	308,4	268,4	313,0	313,0	313,0	308,4	286,9	270,7	279,7
Fragment 2	328,5	311,0	330,4	329,1	331,5	333,4	328,8	329,1	329,2	329,1	373,1	288,5	331,6	308,5
Fragment 3	331,4	331,0	370,1	331,5	333,8	373,1	330,5	331,5	331,6	331,7	447,6	328,8	367,3	330,5
Fragment 4	375,1	359,2	431,1	373,0	373,1	375,5	369,8	348,2	348,1	347,9	449,8	331,1	374,9	370,0
Fragment 5	489,0	428,5	488,8	375,1	375,6	447,4	489,1	375,1	373,0	375,1	457,3	449,7	489,8	388,1
Fragment 6		550,0		417,0	447,5			412,5	375,1	447,5		547,1	494,3	407,1
Fragment 7				447,6	449,7			447,4	447,5			551,8		447,7
Fragment 8				449,7										452,9
Fragment 9				491,5										489,2
Fragment 10														547,0
Fragment 11														549,8
