# Supplementary material for: Recombination Drives Evolution of the Clostridium difficile 16S-23S rRNA Intergenic Spacer Region
Source: PLoS One. 2014 Sep 15;9(9):e106545. doi: 10.1371/journal.pone.0106545 (PMC4164361; doi:10.1371/journal.pone.0106545)
Supplement: Table S2 — List of sequence variants of ISR building blocks. Only the 95 representatives of nun-redundant datasets were taken into account when looking at the variations in ISR sequence building blocks. (PDF) [file pone.0106545.s005.pdf]

Table S2. List of sequence variants of ISR building blocks.

Building block	Sequence variants
Start	AAGGAGAATTACCTACTGTTTAATTTTGA
	AAGGAGAATTGCCTACTGTTTAATTTTGA
	AAGGAGAATTACCTACTGTTCTAATTTTGA
	AAGGGGAATTACCTACTGTTTAATTTTGA
Ntrna	GGGTCCGTTTTTACGAATACTCAAAA
	GGGTTCGTTTGTACGAATACTCAAAA
	GGGTTCGTTTTTACGAATACTCAAAA
	GGGTTCGTTTTTACGAATGCTCAAAA
	GGGTTTGTTTTTACGAATACTCAAAA
Trna	AAGTTCTTTATGAACTTCATATTTGGGGGTGTAGCTCAGTTGGGAGAGCACTTGCCTTGCAAGCAAGGGGTCAGGAGTTC
	GACTCTCCTCATCTCCACCATTTAAGAGCATATTACTTAAATCTTTGATTTATCTAGTAGTCTCTTACATTGCACTTTAAGCTT
	ATTTTTATACAAGCTTGTGTG
	AAGTTCTTTACGAACTTTATATATGGGGGTGTAGCTCAGTTGGGAGAGCACTTGCCTTGCAAGCAAGGGGTCAGGAGTTC
	GACTCTCCTCATCTCCACCATTTAAGAGTATATTACTTAAATCTTTGATTTACTTAGTAGCCTCTTACAATGCACTCATAGCTT
	AAATTTATACAGGCTTTGTGCG
	AAGTTCTTTACGAACTTTATATATGGGGGTGTAGCTCAGTTGGGAGAGCACTTGCCTTGCAAGCAAGGGGTCAGGAGTTC
	GACTCTCCTCATCTCCACCATTTAAGAGTATATTACTTAAATCTTTGATTTACTTAGTAGCCTCTTACAATGCACTTATAGCTT
	AAATTTATACAAGCTTTGTGCG
	AAGTTCTTTACGAACTTTATATATGGGGGTGTAGCTCAGTTGGGAGAGCACTTGCCTTGCAAGCAAGGGGTCAGGAGTTC
	GACTCTCCTCATCTCCACCATTTAAGAGTATATTACTTAAATCTTTGATTTACTTAGTAGCCTCTTACAATGCACTTATAGCTT
	AAATTTATACAGGCTTTGTGCG
	AAGTTTTTTACGAACTTTATATATGGGGGTGTAGCTCAGTTGGGAGAGCACTTGCCTTGCAAGCAAGGGGTCAGGAGTTC
	GACTCTCCTCATCTCCACCATTTAAGAGTATATTACTTAAATCTTTGATTTACTTAGTAGCCTCTTACAATGCACTTATAGCTT
	AAATTTATACAGGCTTTGTGCG
	AAGTTCTTTACGAACTTTATATATGGGGGTGTAGCTCAGTTGGGAGAGCACTTGCCTTGCAAGCAAGGGGTCAGGAGTTC
	GACTCTCCTCATCTCCACCATTTAAGAGTATATTACTTAAATCTTTGATTTACTTAGTAGCCTCTTACAATGCACTTATAGCTT
	AAATTTATACAAGCTTTGTGTG
	AAGTTTTTTACGAACTTTATATATGGGGGTGTAGCTCAGTTGGGAGAGCACTTGCCTTGCAAGCAAGGGGTCAGGAGTTC
	GACTCTCCTCATCTCCACCATTTAAGAATATATTACTTAAATCTTTGATTTACTTAGTAGCCTCTTACAATGCACTTATAGCTT
	AAATTTATACAGGCTTTGTGCG
	AAGTTCTTTACGAACTTTATATATGGGGGTGTAGCTCAGTTGGGAGAGCACTTGCCTTGCAAGCAAGGGGTCAGGAGTTC
	GACTCTCCTCATCTCCACCATTTAAGAGTATATTACTTAAATCTTTGATTTACTTAGTAGCCTCTTACAATGCACTTATAGCTT
	AAATTTATACAGGCTTTGTGTG
	AAGTTCTTTATGAACTTCATATTTGGGGGTGTAGCTCAGTTGGGAGAGCACTTGCCTTGCAAGCAAGGGGTCAGGAGTTC
	GACTCTCCTCATCTCCACCATTTAAGAGTATATTACTTAAATCTTTGATTTACTTAGTAGCCTCTTACAATGCACTTATAGCTT
	AAATTTATACAAGCTTTGTGCG
	AAGTTCTTTATGAACTTCATATTTGGGGGTGTAGCTCAGTTGGGAGAGCACTTGCCTTGCAAGCAAGGGGTCAGGAGTTC
	GACTCTCCTCATCTCCACCATTTAAGAGTGTATTACTTAAATCTTTGATTTACTTAGTAGCCTCTTACAATGCACTTATAGCTT
	AAATTTATACAAGCTTTGTGTG
	AAGTTCTTTACGAACTTTATATTTGGGGGTAGCTCAGTTGGGAGAGCACTTGCCTTGCAAGCAAGGGGTCAGGAGTTCGA
	CTCTCCTCATCTCCACCATTTAAGAGTATATTACTTAAATCTTTGATTTACTTAGTAGCCTCTTACAATGCACTTATAGCTTAA
	ATTTATACAAGCTTTGTGTG
9 bp direct repeat	ATAGCACTT
	CTAGCACTT
	GTAGCACCT
	GTAGCACTT
	TTAGAACTT
	TTAGCACTT
	TTAGTACTT
	TTGGCACTT

Building block	Sequence variants
20 bp	TAAGCAACGGAATTTATTCG
33 bp	TAAGCAACAGAATTTATTCGTTGGCGCCGTGCT
	TAAGCAACAGAATTTATTCGTTGGCGCTGTGCG
	TAAGCAACAGGATTTATTCGTTGGCGCTGTACT
	TAAGCAACAGGATTTATTTGTTGGCGCCGTGCT
	TAAGCAACGGAATTTATTCGTTAGCGCCGTGCT
	TAAGCAACGGAATTTATTCGTTGGCACCGTGCT
	TAAGCAACGGAATTTATTCGTTGGCGACGTGCT
	TAAGCAACGGAATTTATTCGTTGGCGCCGTGCG
	TAAGCAACGGAATTTATTCGTTGGCGCCGTGCT
	TAAGCAACGGAATTTATTCGTTGGCGCTGTGCA
	TAAGCAACGGAATTTATTCGTTGGCGCTGTGCG
	TAAGCAACGGAATTTATTCGTTGGCGCTGTGCT
	TAAGCAACGGAATTTATTCGTTGGCGCTGTGTG
	TAAGCAACGGAATTTATTCGTTGGCGTTGTGCG
	TAAGCAACGGAATTTATTCGTTGGTGCCGTGCT
	TAAGCAACGGAATTTATTTGTTGGCGCCGTGCT
	TAAGCAACGGGATTTATCCGTTGGCGACGTGCT
	TAAGCAACGGGATTTATCCGTTGGCGCCGTGAA
	TAAGCAACGGGATTTATCCGTTGGCGCCGTGCA
	TAAGCAACGGGATTTATCCGTTGGCGCCGTGCT
	TAAGCAACGGGATTTATCCGTTGGCGCTGTGAA
	TAAGCAACGGGATTTATCCGTTGGCGCTGTGCA
	TAAGCAACGGGATTTATCCGTTGGCGCTGTGCG
	TAAGCAACGGGATTTATCCGTTGGCGCTGTGCT
	TAAGCAACGGGATTTATCCGTTGGCGCTGTGTG
	TAAGCAACGGGATTTATTCGTTGGCGACGTGCT
	TAAGCAACGGGATTTATTCGTTGGCGACGTGTT
	TAAGCAACGGGATTTATTCGTTGGCGCCGTGCT
	TAAGCAACGGGATTTATTCGTTGGCGCTGTGCA
	TAAGCAACGGGATTTATTCGTTGGCGCTGTGCG
	TAAGCAACGTGATTTATCCGTTGGCGCTGTGCA
	TAAGCAACGTGATTTATCCGTTGGCGCTGTGTG
	TAAGCAACTGGATTTATCCGTTGGCGCCGTGCT
	TAAGCAATGGAATTTATTCGTTGGCGCTGTGCG
	TAAGCAGCGGGATTTATCCGTTGGCGCCGTGCT
	TAAGCGACGGAATTTATTCGTTGGCGCTGTGCA
	TAGCAACGGGATTTATCCGTTGGCGCCGTGCT
	TTAGCAACGGAATTTATTCGTTGGCGCCGTGCT
	TTAGCAACGGAATTTATTCGTTGGCGCTGTGTG
	TTAGCAACGGAATTTATTCGTTGGCGTTGTGCG
	TTAGCAACGGGATTTATCCGTTGGCGCCGTGCT
	TTAGCAGCGGGATTTATCCGTTGGCGACGTGCT

Building block	Sequence variants
53 bp	TAAGCAACAGAATAAACTAAACGCATGTGAAGTTTGTTTGTTGGCGCTGTGCG
	TAAGCAACAGAATAAACTGAACACATGTGAAGTTTGTTTGTTGGCGCTGTGCG
	TAAGCAACAGAATAAACTGAACACATGTGAAGTTTGTTTGTTGGCGTTGTGCA
	TAAGCAACAGAATAAACTGAACGCATCTGAAGTTTGTTTGTTGGCGCTGTGCG
	TAAGCAACAGAATAAACTGAACGCATGTAAAGTTTGTTTGTTGGCGCTGTGCG
	TAAGCAACAGAATAAACTGAACGCATGTGAAGTTCGTTTGTTGGCGCTGTGTG
	TAAGCAACAGAATAAACTGAACGCATGTGAAGTTTGTTTGTTGGCGCCGTGCG
	TAAGCAACAGAATAAACTGAACGCATGTGAAGTTTGTTTGTTGGCGCTGTGAA
	TAAGCAACAGAATAAACTGAACGCATGTGAAGTTTGTTTGTTGGCGCTGTGCG
	TAAGCAACAGAATAAACTGAACGCATGTGAAGTTTGTTTGTTGGCGCTGTGTG
	TAAGCAACAGAATAAACTGAACGTATGTGAAGTTTGTTTGTTGGCGCTGTGCT
	TAAGCAACAGAATAAACTGAGCGCATGTGAAGTTTGTTTGTTGGCGCTGTGCT
	TAAGCAACAGAATAAACTTAACGCATGTGAAGTTTGTTTGTTGGCGCTGTGCG
	TAAGCAACGGAATAAACTGAACGCATATGAAGTTTGTTTGTTGGCGCTGTGCG
	TAAGCAACGGAATAAACTGAACGCATGTGAAGTTTGTTTGTTGGCATTGTGCG
	TAAGCAACGGAATAAACTGAACGCATGTGAAGTTTGTTTGTTGGCGCTGTGCG
	TAAGCAACGGAATAATCTTAGTGAATACGAAGGTTGTTCGTTGACGTGGTGCA
	TAAGCAACGGAATAATCTTAGTGAATACGAAGGTTGTTCGTTGACGTGGTGCG
	TAAGCAGCAGAATAAACTGAACGCATGTGAAGTTTGTTTGTTGGCGCTGTGTG
	TAAGTAACGAAATAATCTTAGTGAATACGAAGGTTGTTCGTTGACGTGGTGCA
	TAAGTAACGGAATAATCTGAGTGAATACGAAGGTTGTTCGTTGACGCTGTGCG
	TAAGTAACGGAATAATCTGAGTGAATACGAAGGTTGTTCGTTGACGTGGTGCG
	TAAGTAACGGAATAATCTGAGTGAATACGAAGGTTGTTCGTTGACGTGGTGTG
	TGCAACAGAATAAACTGAATGCATGTGAAGTTTGTTTGTTGGCGCTGTGTG
	TTAGCAACAAAATAAACTGAACGCATGTGAAGTTTGTTTGTTAGCGCTGTGCA
	TTAGCAACAGAATAAACTGAACGCATGTGAAGCTTGTTTGTTGGCGCTGTGCG
	TTAGCAACAGAATAAACTGAACGCATGTGAAGTTTGTTTGCTGGCGCTGTGCA
	TTAGCAACAGAATAAACTGAACGCATGTGAAGTTTGTTTGTTGGCGACGTGCT
	TTAGCAACAGAATAAACTGAACGCATGTGAAGTTTGTTTGTTGGCGCCGTGCT
	TTAGCAACAGAATAAACTGAACGCATGTGAAGTTTGTTTGTTGGCGCTGTGCA
	TTAGCAACAGAATAAACTGAACGCATGTGAAGTTTGTTTGTTGGCGCTGTGCG
	TTAGCAACAGAATAAACTGAACGCATGTGAAGTTTGTTTGTTGGCGCTGTGCT
	TTAGCAACAGAATAAACTGAGCGCATGTGAAGTTTGTTTGTTGGCGCTGTGCG
	TTAGCAACAGGATAAACTGAACGCATGTGAAGTTTGTTTGTTGGCGCCGTGCT
	TTAGTAACATAATAAACTGAACGCATGTGAAGTTTGTTTGTTGGCGCTGTGCG

Building block	Sequence variants
End	TGAAAACTGCATATACATTTAGTGATATGACATCTAATTTGTAATATATAAAGCTGATAACTTTTTAAAATTATCGAAGTTGATAGCTTCTAATCTATCAAACCTTTTTAAC
	TGAAAACTGCATATATATATTTAGTGATATGACATCTAATTTGTAATATAAAGCTGATAACTTTTTAAAATTATCGAAGTTGATAGCTTCTAATCTATCAAACCTTTTTAAC
	TGAAAACTGCATATATATATTTAGTGATATGACATCTAATTTGTAATATAAAGCTGATAACTTTTTAAAATTATCGAAGTTGATAGCTTCTAATCTATCAAACCTTTTTAAC
	TGAAAACTGCATATATATATTTAGTGATATGACATCTAATTTGTAATATATAAAGCTGATAACTTTTAAAAATTATCAAGTTGATAGACTTTAATCTATCAAACCTTTTTAAC
	TGAAAACTGCATATATATATTTAGTGATATGACATCTAATTTGTAATATATAAAGCTGATAACTTTTAAAATTATCGAAGTTGATAGCTTCTAATCTATCAAACCTTTTTAAC
	TGAAAACTGCATATATATATTTAGTGATATGACATCTAATTTGTAATATATAAAGCTGATAACTTTTTAAAATTATCGAAGTTGATAGCTTCTAATCTACCAAACCTTTTTAAC
	TGAAAACTGCATATATATATTTAGTGATATGACATCTAATTTGTAATATATAAAGCTGATAACTTTTTAAAATTATCGAAGTTGATAGCTTCTAATCTATCAAACCTTTTTAAC
	TGAAAACTGCATATATATATTTAGTGATATGACATCTAATTTGTAATATATAAAGCTGATAACTTTTTAAAATTATCGAAGTTGATAGCTTCTAATCTATCAAACCTTTTTAAC
	TGAAAACTGCATATATATATTTAGTGATATGACATCTAATTTGTAATATATAAAGCTGATAGCTTTTTAAAATTATCGAAGTTGATAGCTTCTAATCTATCAAACCTTTTTAAC
	TGAAAACTGCATATATATATTTAGTGATATGACATCTAATTTGTAATATATGAAGCTGATAACTTTTTAAAATTATCGAAGTTGATAGCTTCTAATCTATCAAACCTTTTTAAC
	TGAAAACTGCATATATATTTAGTGATATGACATCTAATTTGTAATATACAAAGCTGATAACTTTTTAAAATTATCGAAGTTGATAGCTTCTAATCTATCAAACCTTTTTAAC
	TGAAAACTGCATATATATTTAGTGATATGACATCTAATTTGTAATATACAAAGCTGATAACTTTTTAAAATTATTGAAGTTGATAGCTTCTAATCTATCAAACCTTTTTAAC
	TGAAAACTGCATATATATTTAGTGATATGACATCTAATTTGTAATATATAAAGCTGATAACTTTCTAAAATTATCGAAGTTGATAGCTTCTAATCTATCAAACCTTTTTAAC
	TGAAAACTGCATATATATTTAGTGATATGACATCTAATTTGTAATATATAAAGCTGATAACTTTTAAAAATTATCAAGTTGATAGACTTTAATCTATCAAACCTTTTTAAC
	TGAAAACTGCATATATATTTAGTGATATGACATCTAATTTGTAATATATAAAGCTGATAACTTTTAAAAATTATCAAGTTGATAGACTTTAATCTATCAAACCTTTTTAAC
	TGAAAACTGCATATATATTTAGTGATATGACATCTAATTTGTAATATATAAAGCTGATAACTTTTAAAATTATCGAAGTTGATAGCTTCTAATCTATCAAACCTTTCTCTATATC
	TGAAAACTGCATATATATTTAGTGATATGACATCTAATTTGTAATATATAAAGCTGATAACTTTTAAAATTATCGAAGTTGATAGCTTCTAATCTATCAAACCTTTTTAAC
	TGAAAACTGCATATATATTTAGTGATATGACATCTAATTTGTAATATATAAAGCTGATAACTTTTTAAAATTATCAAAGTTGATAGCTTCTAATCTATCAAACCTTTTTAAC
	TGAAAACTGCATATATATTTAGTGATATGACATCTAATTTGTAATATATAAAGCTGATAACTTTTTAAAATTATCAAATTTAATACTTTCAATCCAATCAAACCTTTTTAA
	TGAAAACTGCATATATATTTAGTGATATGACATCTAATTTGTAATATATAAAGCTGATAACTTTTTAAAATTATCAAGTTGATAGACTTTAATCTATCAAACCTTTTTAAC
	TGAAAACTGCATATATATTTAGTGATATGACATCTAATTTGTAATATATAAAGCTGATAACTTTTTAAAATTATCGAAGTTGATAGCTTCTAATCTATCAAACCTTTTTAAC
	TGAAAACTGCATATATATTTAGTGATATGACATCTAATTTGTAATATATAAAGCTGATAACTTTTTAAAATTATCGAAGTTGATAGCTTCTAATCTATCAAACCTTTTTAAC
	TGAAAACTGCATATATATTTAGTGATATGACATCTAATTTGTAATATATAAAGCTGATAACTTTTTAAAATTATTGAAGTTGATAGCTTCTAATCTATCAAACCTTTTTAAC
	TGAAAACTGCATATATATTTAGTGATATGACATCTAATTTGTAATATATAAAGTTGATAACTTTTTAAAATTATCGAAGTTGATAGCTTCTAATCTATCAAACCTTTTTAAC
	TGAAAACTGCATATATATTTAGTGATATGACATCTAATTTGTAATATATAAAGTTGATAACTTTTTAAAATTATTGAAGTTGATAGCTTCTAATCTATCAAACCTTTTTAAC
	TGAAAACTGCATATATATTTAGTGGTATGACATCTAATTTGTAATATATAAAGCTGATAACTTTTTAAAATTATCGAAGTTGATAGCTTCTAATCTATCAAACCTTTTTAAC
	TGAAAACTGCATATATTTATTTAGTGATATGACATCTAATTTGTAATATATAAAGCTGATAACTTTTAAAAATTATCAAGTTGATAGACTTTAATCTATCAAACCTTTTTAAC
	TGAAAATTGCATATATATATTTAGTGATATGACATCTAATTTGTAATATATAAAGCTGATAACTTTTAAAAATTATCAAGTTGATAGACTTTAATCTATCAAACCTTTTTAAC
	TGAAAATTGCATATATATTTAGTGATATGACATCTAATTTGTAATATACAAAGCTGATAACTTTTTAAAATTATCGAAGTTGATAGCTTCTAATCTATCAAACCTTTTTAAC
	TGAAAATTGCATATATATTTAGTGATATGACATCTAATTTGTAATATACAAAGCTGATAACTTTTTAAAATTATCGAAGTTGATAGCTTCTAATCTATCAAACCTTTTTAAT
	TGAAACTGCATATATATTAGGATATGACATCTAATTTGTATATATAAAGCTGATACTTTTAAAATTATCAGTGATAGCTTTAATCTATCAAACCTTTTTAAC
	TGAAAGCTGCATATATATATTTAGTGATATGACATCTAATTTGTAATATATAAAGCTGATAACTTTTTAAAATTATCGAAGTTGATAGCTTCTAATCTATCAAACCTTTTTAAC
	TGAGAACTGCATATATATATTTAGTGATATGACATCTAATTTGTAATATATAAAGCTGATAACTTTTTAAAATTATCGAAGTTGATAGCTTCTAACCTATCAAACCTTTTTAAC
