# Supplementary material for: Recombination Drives Evolution of the Clostridium difficile 16S-23S rRNA Intergenic Spacer Region
Source: PLoS One. 2014 Sep 15;9(9):e106545. doi: 10.1371/journal.pone.0106545 (PMC4164361; doi:10.1371/journal.pone.0106545)
Supplement: Supporting Information S1 — Sequence identity matrices of 95 representatives of non-redundant sets. (PDF) [file pone.0106545.s007.pdf]

Sequence Identity Matrices

185-188

Seq->	524-9-185	4_B-14
524-9-185	ID	0,984
4_B-14	0,984	ID

217-219

Seq->	8_A-6	8_176_3	012-16	029-19	434-15
8_A-6	ID	0,964	0,977	0,986	0,968
8_176_3	0,964	ID	0,946	0,964	0,946
012-16	0,977	0,946	ID	0,973	0,964
029-19	0,986	0,964	0,973	ID	0,973
434-15	0,968	0,946	0,964	0,973	ID

238-240

Seq->	078-1	247-5	519-10	524-10
078-1	ID	0,966	0,979	0,966
247-5	0,966	ID	0,987	0,974
519-10	0,979	0,987	ID	0,987
524-10	0,966	0,974	0,987	ID

260-263

Seq->	12_AT-16	19_AT-3	012-4	012-11	045-1	066.2	251-19	484-3	524-20
12_AT-16	ID	0,966	0,936	0,933	0,94	0,937	0,922	0,922	0,944
19_AT-3	0,966	ID	0,969	0,966	0,973	0,97	0,955	0,955	0,977
012-4	0,936	0,969	ID	0,969	0,973	0,966	0,962	0,977	0,984
012-11	0,933	0,966	0,969	ID	0,97	0,955	0,955	0,962	0,984
045-1	0,94	0,973	0,973	0,97	ID	0,973	0,973	0,973	0,981
066.2	0,937	0,97	0,966	0,955	0,973	ID	0,973	0,966	0,97
251-19	0,922	0,955	0,962	0,955	0,973	0,973	ID	0,962	0,97
484-3	0,922	0,955	0,977	0,962	0,973	0,966	0,962	ID	0,977
524-20	0,944	0,977	0,984	0,984	0,981	0,97	0,97	0,977	ID

279-282

Seq->	24_AT-17	2_AI5_8	23_B-12	012-8	012-12	027-R	029-15	029-17	033-11	033-13	063.1-4	063.1-11	063-1	063-10	126-17	247-10	247-15	434-12	444-15	0451-2	519-13	519-14	AI56-7	PR124-6
24_AT-17	ID	0,982	0,971	0,833	0,975	0,985	0,972	0,982	0,975	0,982	0,965	0,829	0,978	0,839	0,972	0,964	0,978	0,979	0,842	0,975	0,985	0,982	0,835	0,968
2_AI5_8	0,982	ID	0,961	0,833	0,979	0,982	0,975	0,964	0,958	0,965	0,947	0,829	0,968	0,832	0,954	0,954	0,961	0,968	0,835	0,965	0,985	0,978	0,828	0,951
23_B-12	0,971	0,961	ID	0,805	0,954	0,978	0,965	0,975	0,958	0,961	0,947	0,802	0,971	0,825	0,951	0,964	0,971	0,979	0,828	0,954	0,964	0,975	0,821	0,944
012-8	0,833	0,833	0,805	ID	0,836	0,826	0,82	0,822	0,829	0,829	0,815	0,982	0,812	0,968	0,826	0,809	0,815	0,826	0,961	0,833	0,829	0,822	0,975	0,819
012-12	0,975	0,979	0,954	0,836	ID	0,975	0,968	0,958	0,965	0,972	0,958	0,833	0,961	0,822	0,961	0,947	0,954	0,975	0,826	0,972	0,979	0,972	0,819	0,961
027-R	0,985	0,982	0,978	0,826	0,975	ID	0,986	0,982	0,965	0,968	0,954	0,822	0,985	0,846	0,958	0,971	0,978	0,986	0,849	0,975	0,985	0,996	0,842	0,958
029-15	0,972	0,975	0,965	0,82	0,968	0,986	ID	0,968	0,951	0,954	0,94	0,816	0,972	0,832	0,944	0,958	0,965	0,972	0,836	0,961	0,975	0,982	0,829	0,944
029-17	0,982	0,964	0,975	0,822	0,958	0,982	0,968	ID	0,968	0,972	0,958	0,819	0,975	0,835	0,961	0,968	0,982	0,982	0,839	0,965	0,968	0,978	0,839	0,954
033-11	0,975	0,958	0,958	0,829	0,965	0,965	0,951	0,968	ID	0,979	0,982	0,829	0,958	0,826	0,989	0,954	0,965	0,979	0,829	0,982	0,961	0,961	0,822	0,979
033-13	0,982	0,965	0,961	0,829	0,972	0,968	0,954	0,972	0,979	ID	0,968	0,826	0,961	0,822	0,982	0,954	0,968	0,982	0,826	0,972	0,968	0,965	0,819	0,965
063.1-4	0,965	0,947	0,947	0,815	0,958	0,954	0,94	0,958	0,982	0,968	ID	0,815	0,951	0,815	0,979	0,944	0,954	0,968	0,819	0,979	0,951	0,951	0,812	0,989
063.1-11	0,829	0,829	0,802	0,982	0,833	0,822	0,816	0,819	0,829	0,826	0,815	ID	0,809	0,951	0,826	0,805	0,812	0,822	0,951	0,833	0,826	0,819	0,958	0,819
063-1	0,978	0,968	0,971	0,812	0,961	0,985	0,972	0,975	0,958	0,961	0,951	0,809	ID	0,839	0,951	0,964	0,971	0,979	0,842	0,961	0,971	0,982	0,835	0,954
063-10	0,839	0,832	0,825	0,968	0,822	0,846	0,832	0,835	0,826	0,822	0,815	0,951	0,839	ID	0,819	0,828	0,835	0,833	0,985	0,833	0,832	0,842	0,992	0,819
126-17	0,972	0,954	0,951	0,826	0,961	0,958	0,944	0,961	0,989	0,982	0,979	0,826	0,951	0,819	ID	0,947	0,958	0,972	0,822	0,982	0,958	0,954	0,815	0,975
247-10	0,964	0,954	0,964	0,809	0,947	0,971	0,958	0,968	0,954	0,954	0,944	0,805	0,964	0,828	0,947	ID	0,971	0,972	0,832	0,951	0,961	0,975	0,825	0,94
247-15	0,978	0,961	0,971	0,815	0,954	0,978	0,965	0,982	0,965	0,968	0,954	0,812	0,971	0,835	0,958	0,971	ID	0,979	0,839	0,961	0,964	0,975	0,832	0,951
434-12	0,979	0,968	0,979	0,826	0,975	0,986	0,972	0,982	0,979	0,982	0,968	0,822	0,979	0,833	0,972	0,972	0,979	ID	0,836	0,975	0,972	0,982	0,829	0,965
444-15	0,842	0,835	0,828	0,961	0,826	0,849	0,836	0,839	0,829	0,826	0,819	0,951	0,842	0,985	0,822	0,832	0,839	0,836	ID	0,836	0,835	0,846	0,985	0,822
0451-2	0,975	0,965	0,954	0,833	0,972	0,975	0,961	0,965	0,982	0,972	0,979	0,833	0,961	0,833	0,982	0,951	0,961	0,975	0,836	ID	0,968	0,972	0,829	0,982
519-13	0,985	0,985	0,964	0,829	0,979	0,985	0,975	0,968	0,961	0,968	0,951	0,826	0,971	0,832	0,958	0,961	0,964	0,972	0,835	0,968	ID	0,985	0,828	0,954
519-14	0,982	0,978	0,975	0,822	0,972	0,996	0,982	0,978	0,961	0,965	0,951	0,819	0,982	0,842	0,954	0,975	0,975	0,982	0,846	0,972	0,985	ID	0,839	0,954
AI56-7	0,835	0,828	0,821	0,975	0,819	0,842	0,829	0,839	0,822	0,819	0,812	0,958	0,835	0,992	0,815	0,825	0,832	0,829	0,985	0,829	0,828	0,839	ID	0,815
PR124-6	0,968	0,951	0,944	0,819	0,961	0,958	0,944	0,954	0,979	0,965	0,989	0,819	0,954	0,819	0,975	0,94	0,951	0,965	0,822	0,982	0,954	0,954	0,815	ID

321-324

Seq->	11_078_11	30_AT-7	31_AT-9	32_AT-8	033-10	033-12	063-12	251-11	441-2	542-8	CD196
11_078_11	ID	0,83	0,833	0,827	0,981	0,969	0,824	0,987	0,827	0,836	0,948
30_AT-7	0,83	ID	0,981	0,972	0,842	0,845	0,966	0,836	0,963	0,969	0,827
31_AT-9	0,833	0,981	ID	0,966	0,845	0,842	0,978	0,839	0,975	0,975	0,835
32_AT-8	0,827	0,972	0,966	ID	0,839	0,83	0,975	0,833	0,978	0,984	0,836
033-10	0,981	0,842	0,845	0,839	ID	0,984	0,836	0,987	0,842	0,848	0,957
033-12	0,969	0,845	0,842	0,83	0,984	ID	0,833	0,975	0,83	0,839	0,948
063-12	0,824	0,966	0,978	0,975	0,836	0,833	ID	0,83	0,978	0,978	0,835
251-11	0,987	0,836	0,839	0,833	0,987	0,975	0,83	ID	0,833	0,842	0,954
441-2	0,827	0,963	0,975	0,978	0,842	0,83	0,978	0,833	ID	0,981	0,829
542-8	0,836	0,969	0,975	0,984	0,848	0,839	0,978	0,842	0,981	ID	0,827
CD196	0,948	0,827	0,835	0,836	0,957	0,948	0,835	0,954	0,829	0,827	ID

342-344

Seq->	29_AT-11	34_AT-14	029-7	444-6	542-13
29_AT-11	ID	0,968	0,945	0,83	0,936
34_AT-14	0,968	ID	0,977	0,862	0,968
029-7	0,945	0,977	ID	0,851	0,985
444-6	0,83	0,862	0,851	ID	0,857
542-13	0,936	0,968	0,985	0,857	ID

362-366

Seq->	066-7	078-4	247-18	542-22
066-7	ID	0,978	0,948	0,836
078-4	0,978	ID	0,951	0,844
247-18	0,948	0,951	ID	0,845
542-22	0,836	0,844	0,845	ID

377-380

Seq->	029-1	484-2	CD196-3	7_176_4
029-1	ID	0,945	0,953	0,96
484-2	0,945	ID	0,976	0,984
CD196-3	0,953	0,976	ID	0,992
7_176_4	0,96	0,984	0,992	ID

383-384

Seq->	444-3	35_AT-12	36_AT-1
444-3	ID	0,969	0,979
35_AT-12	0,969	ID	0,989
36_AT-1	0,979	0,989	ID

399-400

Seq->	027-4	033-1	519-1	542-12
027-4	ID	0,987	0,97	0,962
033-1	0,987	ID	0,967	0,955
519-1	0,97	0,967	ID	0,953
542-12	0,962	0,955	0,953	ID

439-442

Seq->	39_A-15	41_B-11	012-4	063.1	126-10	247-11	444-7	542-29
39_A-15	ID	0,986	0,984	0,964	0,957	0,97	0,973	0,946
41_B-11	0,986	ID	0,975	0,977	0,97	0,984	0,986	0,959
012-4	0,984	0,975	ID	0,97	0,964	0,973	0,984	0,953
063.1	0,964	0,977	0,97	ID	0,984	0,984	0,986	0,946
126-10	0,957	0,97	0,964	0,984	ID	0,977	0,979	0,944
247-11	0,97	0,984	0,973	0,984	0,977	ID	0,984	0,957
444-7	0,973	0,986	0,984	0,986	0,979	0,984	ID	0,959
542-29	0,946	0,959	0,953	0,946	0,944	0,957	0,959	ID

501-504

Seq->	30_001_11	029-5	434-13	434-15	441-3	519-7	630-1
30_001_11	ID	0,986	0,98	0,982	0,98	0,976	0,978
029-5	0,986	ID	0,986	0,984	0,986	0,978	0,984
434-13	0,98	0,986	ID	0,982	0,98	0,972	0,974
434-15	0,982	0,984	0,982	ID	0,978	0,982	0,972
441-3	0,98	0,986	0,98	0,978	ID	0,972	0,974
519-7	0,976	0,978	0,972	0,982	0,972	ID	0,966
630-1	0,978	0,984	0,974	0,972	0,974	0,966	ID
